# Absence of TSC1 Accelerates CD8^+^ T cell-mediated Acute Cardiac Allograft Rejection

**DOI:** 10.14336/AD.2022.0224

**Published:** 2022-10-01

**Authors:** Liang Tan, Yanan Xu, Gongbin Lan, Hongxia Wang, Zhanfeng Liang, Zhaoqi Zhang, Qianchuan Tian, Yangxiao Hou, Yong Zhao, Xubiao Xie

**Affiliations:** ^1^Department of Kidney Transplantation, Second Xiangya Hospital of Central South University, Changsha, China; ^2^Clinical Research Center for Organ Transplantation in Hunan Province, Changsha, China.; ^3^State Key Laboratory of Membrane Biology, Institute of Zoology, Chinese Academy of Sciences, University of Chinese Academy of Sciences, Beijing, China.; ^4^Laboratory Medicine Center, Nanfang Hospital, Southern Medical University, Guangzhou, Guangdong, China; ^5^Institute for Stem Cell and Regeneration, Chinese Academy of Sciences, Beijing, China.

**Keywords:** tuberous sclerosis complex 1, acute rejection, cardiac transplantation

## Abstract

Tuberous sclerosis complex (TSC) is an autosomal dominant disease caused by inactivating mutations in *TSC1* or *TSC2*.Patients with TSC often require organ transplantation after organ failure. TSC1 serves as an important control node in immune cell development and responses; however, its effect on T cells in transplant immunity has not yet been explored. Here, we characterized the effect of TSC1 deficiency in T cells on acute allograft rejection using a mouse cardiac transplantation model. We observed compromised allograft survival in mice with TSC1-deficient T cells. Notably, the allografts in mice transferred with TSC1-deficient CD8^+^T cells showed accelerated acute allograft rejection. TSC1 deficiency triggered the increased accumulation of CD8^+^ T cells in allografts due to augmented infiltration caused by increased CXCR3 expression levels and elevated in-situ proliferation of TSC1-deficient CD8^+^ T cells. Compared to CD8^+^ T cells from wild-type (WT) mice, TSC1-deficient CD8^+^ T cells exhibited enhanced cell proliferation and increased expression levels of interferon-γ and granzyme B after alloantigen stimulation. Rapamycin, an inhibitor of mammalian target of rapamycin (mTOR), is used to treat patients with TSC and prevent rejection after solid-organ transplantation. Although rapamycin induced most cardiac allografts to survive beyond 100 d in WT mice, rapamycin-treated cardiac allografts in TSC1-deficient mice were rejected within 60 d. These results suggest that TSC1-deficient recipients may be more resistant to rapamycin-mediated immunosuppression during organ transplantation. Collectively, TSC1 significantly accelerates acute allograft rejection by enhancing the alloreactivity of CD8^+^ T cells, making them more resistant to mTOR inhibitor-mediated immunosuppression.

Organ transplantation is the only effective treatment for patients with end-stage organ failure [1, 2]. T cells play a pivotal role in transplant rejection, which is a major barrier to the long-term survival of allografts[3]. Mammalian target of rapamycin (mTOR), including mTOR complex 1 (mTORC1) and 2 (mTORC2), has various functions in different T cell subpopulations [4-6]. It is well known that functional mutations of tuberous sclerosis complex 1(*TSC-1*), a negative regulator of mTOR, can cause TSC, and the affected patients may require organ transplantation after organ failure [7-12]. However, the role of TSC1 in T cells in transplant immunity remains unclear.

Previously, we demonstrated that TSC1 regulates macrophage polarization, thymic epithelial cell survival and development, thymic regulatory T cell (Treg) development, and CD8^+^ T cell survival and homeostasis [13-16]. The expression levels of TSC1 in activated CD8^+^ T cells are higher than those in B and CD4^+^ T cells [13]. We and others have demonstrated that TSC1 deficiency reduces the number of peripheral CD8^+^ T cells, while increasing that of activated/memory of T cells in primary mice [13, 17, 18]. TSC1-deficient T cells, especially CD8^+^ T cells, have higher caspase activity and display impaired survival capacity [13, 17-19]. Although TSC1 deficiency enhances mTORC1 activation in T cells, mTORC1 inhibitor, rapamycin, does not improve the survival rate of TSC1-deficient T cells [13, 18]. TSC1 deficiency impairs the antigen-specific responses of CD8^+^ T cells in the bacteria-infected mice, causing these cells to exhibit decreased antigen-reactivity, amplification and interferon (IFN)-γ production [17, 20, 21]. However, the effects of TSC1 on T cells have been inconsistent. We have previously shown that TSC1 deficiency does not change the number of splenic CD4^+^ T cells [13], whereas others have found that TSC1 deficiency significantly decreases the number of splenic CD4^+^ T cells [17, 18]. In addition, one study suggests that TSC1 deficiency enhances T cell proliferation [18], whereas others reveal that TSC1 deficiency does not affect the proliferation of CD4^+^ or CD8^+^T cells triggered by T cell receptor (TCR) activation and costimulatory signaling [22].

We herein demonstrate that TSC1 deficiency enhances the alloreactivity of CD8^+^T cells by enhancing the graft-accumulation ability, proliferation, and effector molecule production of alloreactive CD8^+^T cells, thereby accelerating acute allograft rejection. In addition, TSC1- deficient T cells impair the effect of mTORC1 inhibitor, rapamycin, on allograft survival.

## MATERIALS AND METHODS

### Mice

We purchased 6-12-week-old C57BL/6(H-2b) and BALB/c(H-2d) male mice from Beijing SPF Biotechnology Co., Ltd. Lck-Cre^+^TSC1^loxp/loxp^ (hereafter referred to as TSC1^-/-^) mice were generated as previously described [13]. All animals were maintained in microisolator cages in specific-pathogen-free facilities. All procedures and protocols were approved by the Institutional Animal Care and Use Committee of the Institute of Zoology.

### Transplantation and Treatment

We performed the mouse cervical cardiac transplantation as previously described [23]. We monitored the cardiac graft pulsation daily and confirmed that the day of rejection was the day of complete cessation of cardiac contractility via laparotomy. To demonstrate the effect of rapamycin on allograft survival, we dissolved rapamycin in 0.2% carboxymethyl cellulose sodium (CMC) solution and injected the recipient mice intraperitoneally with rapamycin (1 mg/kg/day) (Cat#: 37094-10MG, Sigma), or CMC from day 0 to 13 after transplantation.

### Graft-infiltrated Cell Isolation

We sacrificed the recipient mice and perfused the allografts with 5 mL of 0.9% NaCl solution containing 125 U/mL heparin sodium in-situ. The allografts were removed, chopped into pieces, and digested at 37 °C for 45 min in RPMI 1640 medium containing 300 U/mL type Ⅱ collagenase (Cat#: LS004176; Worthington) and 20 U/ml DNase Ⅰ (Cat#: D5025-150KU; Sigma). The cell suspension was passed through a 200-gauge stainless steel mesh.

### Monoclonal antibodies and Chemical Reagents

The following fluorochrome-conjugated monoclonal antibodies (mAbs) were used in this study: anti-mCD4-PE (clone GK1.5, Cat#: 100408; BioLegend), anti-mCD4-PE-Cy5 (clone GK1.5, Cat#:100410; BioLegend), anti-mCD4-APC (clone GK1.5, Cat#:100412; Biolegend), anti-mCD4-APC-Cy7 (clone GK1.5, Cat#: 100412; BioLegend), anti-mCD44-fluorescein isothiocyanate (FITC) (clone IM7, Cat#: 103006; BioLegend), anti-mCD44-APC (clone IM7, Cat#: 559250; BD Pharmingen), anti-mGr-1-FITC (clone RB6-8C5, Cat#: 108451; BioLegend), anti-mIFN-γ-PE (clone XMG1.2, Cat#: 505808; BioLegend), anti-mF4/80-FITC (clone BM8, Cat#: 11-4801-82; eBioscience), anti-mCD8a-PE (clone 53-6.7, Cat#: 100725; BioLegend), anti-mCD8a-PE-Cy5 (clone 53-6.7, Cat#: 100710; BioLegend), anti-mCD45-PE-Cy5 (clone 30-F11, Cat#: 103132; BioLegend), anti-mCD45-PE-Cy7 (clone 30-F11, Cat#: 103114; BioLegend), anti-mCD45-APC (clone 30-F11, Cat#: 103112; BioLegend), anti-mTCR-β-PE-Cy7 (clone H57-597, Cat#: 109222; BioLegend), anti-mCD62L-FITC (clone MEL-14, Cat#: 11-0621-82; eBioscience), anti-mCD62L-PE (clone MEL-14, Cat#: 12-0621-82; eBioscience), anti-Foxp3-FITC (clone FJK-16s, Cat#: 11-5773-82; eBioscience), anti-mCD11b- PE-Cy5 (clone M1/70, Cat#: 15-0112-83; eBioscience), anti-mGranzyme B-FITC (clone NGZB, Cat#: 11-8898-80; eBioscience), anti-mCD11c-PE (clone N418, Cat#: 12-0114-82; eBioscience), anti-mouse C-X-C chemokine receptor type 3 (CXCR3)-FITC (clone CXCR3-173, Cat#: 11-1831-80; eBioscience), and anti-T-bet-PE (clone 4B10, Cat#:12-5825-82; eBioscience). We obtained FcR-mAb (clone 2.4 G2, Cat#: 553141), GolgiStop (Cat#: 554724), and Mouse Anti-Ki-67 set (Cat#: 556027) from BD Pharmingen, ionomycin (Cat#: I9657), phorbol 12-myristate 13-acetate (PMA; Cat#: P1585) and mitomycin C (Cat#: M0503) from Sigma-Aldrich, and 5(6)-carboxyfluorescein diacetate succinimidyl ester (CFSE; MCE, HY-D0938) from MedChemExpress.

### Flow Cytometry

Flow cytometric analysis was performed as previously described [24, 25]. Briefly, single-cell suspensions of spleen and heart were prepared, and Fc-receptors of sample cells were blocked by incubation with anti-Fc receptor antibodies (2.4G2) for 15 min before staining with the indicated antibodies. All the indicated antibodies were checked by a single stain to validate the antibody specificity. For multicolor flow cytometry experiments, fluorescence minus one control (including anti-CXCR3 and anti-granzyme B stains) was employed to distinguish the genuine target from the background. For membrane surface protein staining, we stained the cells with the flow cytometry antibodies for 0.5 h at 4 °C. For cytokine staining, the cells were stimulated with 50 ng/mL PMA and 500 ng/mL ionomycin and GolgiStop for 5 h at 37°C before staining. For nuclear protein staining or intracellular protein staining, we fixed the cells with fixation/permeabilization buffer solution from BD Biosciences or eBioscience, following the manufacturer’s protocol after membrane surface protein staining. We performed flow cytometry analysis using the Beckman Coulter Epice XL(Beckman), Gallios (Beckman) or Fluorochrome/Laser reference poster (BD Biosciences).

### Adoptive Transfer Experiment

We purified CD8^+^T cells from the peripheral lymph nodes and spleens of primary wild-type (WT) mice or TSC1^-/-^ mice using the Mojosort Mouse CD8^+^T Cell Isolation Kit (Cat#: 480035; BioLegend). The purity of enriched CD8^+^T cells was > 97%. To detect whether TSC1 deficiency enhanced the role of CD8^+^T cells in acute allograft rejection, we adoptively transferred 2.0 × 10^6^ isolated cells into WT mice, who accepted cardiac allografts within 24 h after adoptive transfer. To define the allograft-infiltrating ability of CD8^+^ T cells, we labelled the isolated cells with CFSE and adoptively transferred 1.7 to 2.0 × 10^6^ cells into WT mice, who accepted the cardiac allografts within 24 h after adoptive transfer. We analyzed the allograft and splenic CD8^+^ CFSE^+^T cells at day 3 after transplantation.

### Real-Time Polymerase Chain Reaction Assay

Total RNA was extracted from allografts on day 4 post-transplantation using Trizo Reagent (Ref: 15596018; Ambion) and the first-strand cDNAs were reverse transcribed. Real-time polymerase chain reaction (PCR) was performed on a CFX96 Real-Time System (Bio-Rad Laboratories, Hercules, CA, USA) using the Power SYBR PCR Master Mix (Applied Biosystems, Foster City, CA, USA). Gene expression relative to mRNA expression in the allografts of WT mice was analyzed using the 2^-ΔΔCt^ method. The primer pairs used for amplification are summarized in Table 1.

**Table 1:** Primer for RT-PCR.

Primer	Nucleotide sequence
**IFN-γ-Forward**	5’-GAACTGGCAAAAGGATGGTGA-3’
**IFN-γ-Reverse**	5’-TGTGGGTTGTTGACCTCAAAC-3’
**Perforin-Forward**	5’-ATCCGACAGTGGCGTCTTGGT-3’
**Perforin-Reverse**	5’-TGACCGAGTGGCAGTGTAGCA-3’
**Granzyme B-Forward**	5’-CCTCCAGGACAAAGGCAG-3’
**Granzyme B-Reverse**	5’-CAGTCAGCACAAAGTCCTCTC-3’
**CXCR3-Forward**	5’-CAAGTTCCCAACCACAAGTG-3’
**CXCR3-Reverse**	5’-AGAAAGGCAAAGTCCGAGG-3’
**HPRT-Forward**	5’-AGTACAGCCCCAAAATGGTTAAG-3’
**HPRT-Reverse**	5’-CTTAGGCTTTGTATTTGGCTTTTC-3’

### Mixed Lymphocyte Reaction

Splenocytes from primary WT or TSC1^-/-^ mice were used as responder cells, while those from BALB/c mice were used as stimulator cells. We labelled the response cells with CFSE (5 μM) for 10 min at 37 °C and pretreated the stimulator cells with mitomycin C (50 μg/mL) for 20 min at 37 °C. We co-cultured the responder (5 × 10^5^ cells per well) and stimulator (2.5 × 10^5^ cells per well) cells in RPMI 1640 medium containing 10% fetal bovine serum, 2 mM L-glutamine, 100 IU/mL penicillin, 100 μg/mL streptomycin, and 50 μM/mL 2-mercaptoethanol in a 96-well U-bottomed plates at 37 °C and 5% CO_2_ for 72 h.

### Enzyme-linked Immunosorbent Assay Measurement

To analyze the serum levels of IFN-γ, we extracted serum samples from recipient mice and stored them at -80 °C. We then determined IFN-γ levels via the enzyme-linked immunosorbent assay (ELISA) kit (Cat#: 430802; BioLegend), following the manufacturer instructions.

### Histology

The cardiac allografts were fixed in formaldehyde and embedded in paraffin. The tissues were cut into 5 μm sections, followed by baking, deparaffinizing, rehydration, and hematoxylin and eosin (H&E) staining. We evaluated the tissue sections according to the standardization guidelines of International Society for Heart and Lung Transplantation (ISHLT) [26].

### Statistical Analysis

Data were analyzed using SPSS 22 and GraphPad Prism 7 software. All data are presented as the mean ± standard error of the mean. Graft survival was analyzed using the log-rank test. Normal distribution and homogeneity of variance of data were analyzed using the Kolmogorov-Smirnov and F-value tests, respectively. Based on the assumptions of a normal distribution and variance homogeneity test, we analyzed ki67 expressions using the paired Student’s *t*-test and compared other quantitative data using Student’s *t*-test or Mann-Whitney U test. Statistical significance was set at *P* < 0.05.

**Figure 1.**
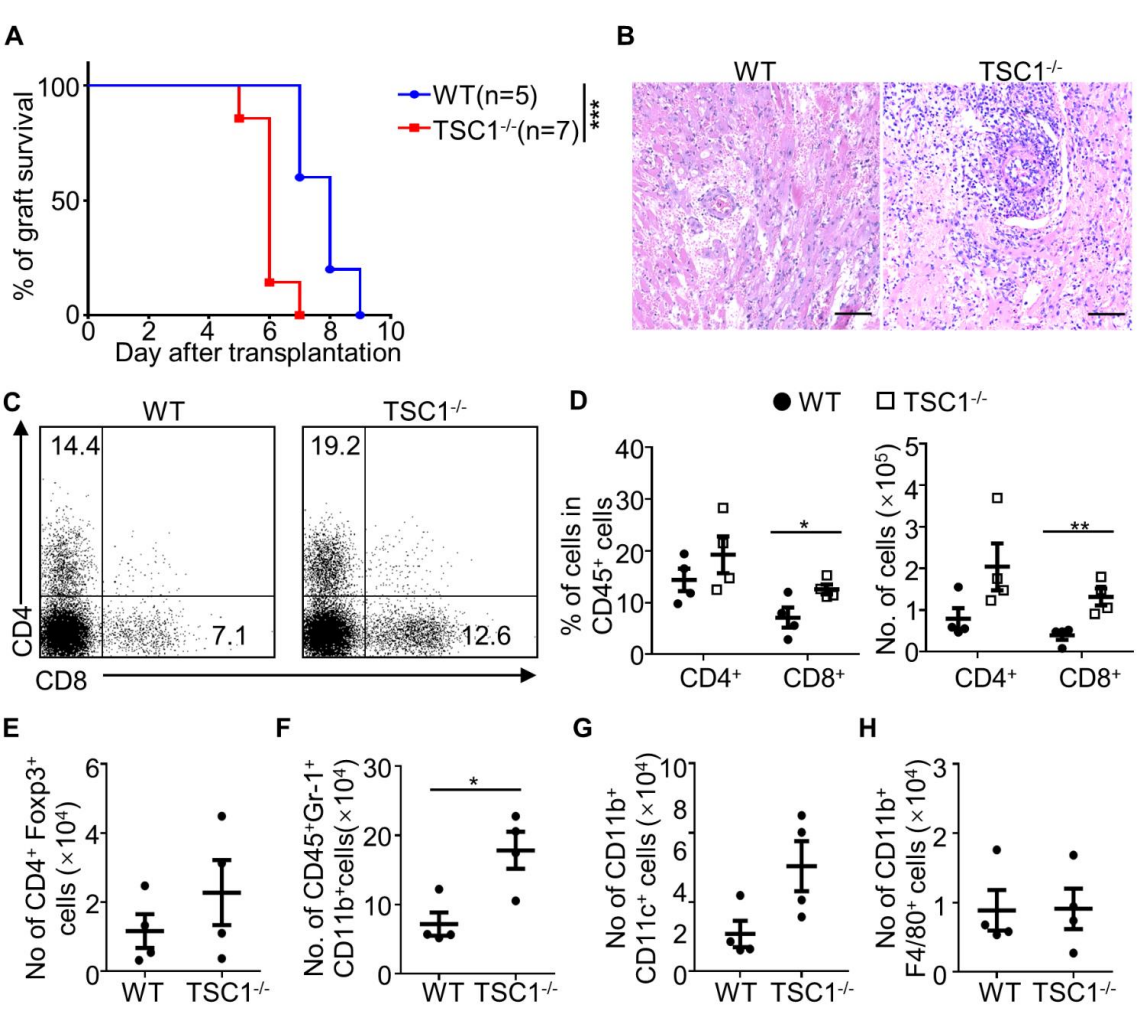
**TSC1 deficiency in T cells decreases the survival of cardiac allografts and enhances CD8^+^ T cell accumulation in allografts**. (**A**) Fully major histocompatibility complex (MHC)-mismatched cardiac allografts from BALB/c mice were transplanted into WT or TSC1^-/-^ mice, and the survival of cardiac allografts was analyzed. Log-rank test; ***P < 0.005, n = 5-7 per group. (**B**) We determined the allograft rejection using histology with hematoxylin and eosin staining (B). Scale bar: 200 μm. We harvested some cardiac allografts on day 4 post-transplantation and analyzed the cell number of the infiltrated CD4^+^ and CD8^+^ (C and D), CD4^+^Foxp3^+^ (E), CD45^+^Gr-1^+^ (F), CD11b^+^ CD11c^+^ (G), and CD11b^+^ F4/80^+^ (H) cells. Student’s *t*-test for (D, E, F, and H) and Mann-Whitney U test for (G); *P < 0.05, **P < 0.01, n = 4 per group.

**Figure 2.**
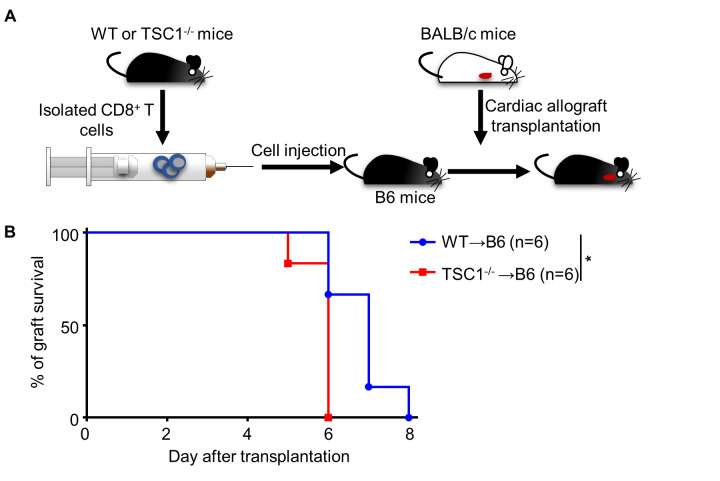
**TSC1-deficient CD8^+^ T cells accelerate the acute allograft rejection**. We isolated and infused TSC^-/-^ (WT→TSC^-/-^) or WT CD8^+^ (WT→WT) T cells into B6 recipient mice (2 × 10^6^ cells per mouse) within 24 h pre-transplantation (A) and analyzed the survival of cardiac allografts (B). Log-rank test; * *P* < 0.05, n = 6 per group.

## RESULTS

### T cell-specific TSC1 deficiency accelerates acute rejection and enhances CD8^+^ T cell allograft -accumulation

Consistent with previous results[13], the number of peripheral CD8^+^T cells was decreased, and the ratio of CD4^+^T cells to CD8^+^T cells was higher in TSC1^-/-^ mice than in WT mice (Supplementary Fig. 1). To evaluate the effect of T cells on graft rejection, cardiac allografts were transplanted into WT and TSC1^-/-^ mice. The results showed that the survival rates of allografts were significantly shorter in TSC1^-/-^ mice (6.00 ± 0.22 d) than in WT mice (7.80 ± 0.37 d; Fig. 1A). Moreover, allografts from WT and TSC1^-/-^ mice showed inflammatory accumulation and myocyte damage (Fig. 1B). Therefore, TSC1 deficiency in T-cells is detrimental to allograft survival.

To further explore the involvement of TSC1-deficient T cells in acute allograft rejection, we obtained allografts on day 4 after transplantation and determined the number of allograft-infiltrated immune cells in them. The percentage and number of allograft-infiltrated CD8^+^ T cells, but not CD4^+^T cells, were significantly higher in TSC1^-/-^ recipient mice than in WT mice (Fig. 1C, D). The ratio of allograft-infiltrated CD8^+^ T cells to CD4^+^ T cells was increased from 0.49 in WT mice to 0.64 in TSC1^-/-^ mice. TSC1 deficiency did not affect the number of allograft-infiltrated CD4^+^Foxp3^+^ cells (Fig. 1E), suggesting that accelerated acute allograft rejection in TSC1^-/-^ mice was not due to Tregs. Consistent with the accelerated cardiac allograft rejection, the number of allograft-accumulated CD11b^+^Gr-1^+^ neutrophils (Fig. 1F) was significantly higher in TSC1^-/-^ mice than in WT mice; however, the number of CD11b^+^CD11c^+^ dendritic cells (Fig. 1G) and CD11b^+^F4/80^+^ macrophages was not affected (Fig. 1H). With respect to the adverse effects of allograft-infiltrated neutrophils on allografts [26-28], increased neutrophil allograft accumulation represented enhanced allograft rejection in TSC1^-/-^ mice. Therefore, increased inflammatory immune cell infiltration in the cardiac allografts of TSC1^-/-^ mice indicates that TSC1-deficienct T cells in TSC1^-/-^ mice display accelerated and severe cell-mediated acute allograft rejection.

**Figure 3.**
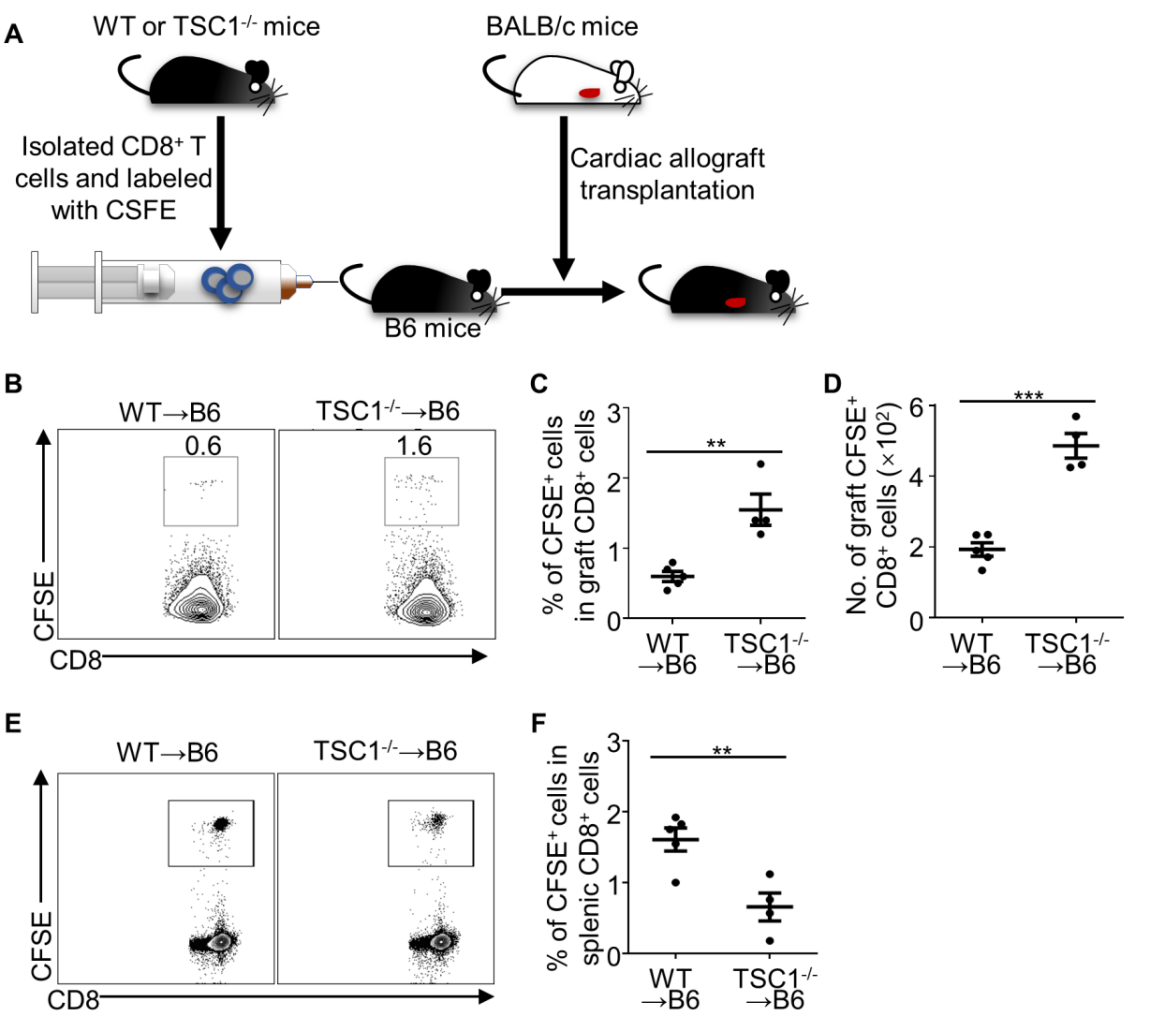
**TSC1-deficiency promotes the allograft-infiltration of CD8^+^ T cells**. We isolated TSC^-/-^ or WT CD8^+^ T cells, labeled them with CFSE, and infused them into B6 recipient mice (2 × 10^6^ cells per mouse) within 24 h before cardiac allograft transplantation (A). We harvested the cardiac allografts and spleens on day 3 post-transplantation and analyzed the inner-graft CFSE^+^ cells (B, C, and D) and splenic CFSE^+^ cells (E). Student’s *t*-test; *P < 0.05, **P < 0.01, ***P < 0.005, n = 4 per group.

### TSC1 deficiency enhances cell-mediated -rejection and allograft -accumulation capacities of CD8^+^ T cells

Increased number of allograft-infiltrated CD8^+^ T cells in TSC1^-/-^ mice prompted us to consider whether CD8^+^ T cells were responsible for the enhanced allograft rejection in TSC1^-/-^ mice. To verify whether TSC1-deficient CD8^+^ T cells accelerated acute allograft rejection, we adoptively transferred CD8^+^ T cells fromWT and TSC1^-/-^ mice into syngeneic WT mice before transplantation (Fig. 2A). Compared to the allografts in mice with WT CD8^+^ T cell adoptive transfer (6.83 ± 0.37 d), those in mice with TSC1-deficient CD8^+^ T cell adoptive transfer exhibited shorter survival rates (5.83 ± 0.17 d; Fig. 2B). These results indicate that TSC1-deficient CD8^+^ T cells exhibit enhanced abilities to mediate acute allograft rejection.

As the accumulation of CD8^+^T cells in allografts is attributed to infiltration and in situ proliferation of cells[29, 30], we aimed to determine the effect of TSC1 on the allograft-infiltrating abilities of CD8^+^ T cells. CD8^+^ T cells from WT and TSC1^-/-^ mice were isolated and labeled with CFSE, and then adoptively transferred into WT mice following by allograft transplantation (Fig. 3A). On day 3 post-transplantation, the percentage and number of allograft-infiltrated TSC1^-/-^ CFSE^+^CD8^+^ T cells were significantly higher than those of WT CFSE^+^CD8^+^ T cells (Fig. 3B-3D). Meanwhile, we found that the percentage of TSC1^-/-^ CFSE^+^ splenic CD8^+^T cells was lower than that of WT CFSE^+^ cells (Fig. 3E, 3F). This suggests that TSC1 deficiency promotes CD8^+^ T cell infiltration into allografts, but not into peripheral lymphatic organs.

**Figure 4.**
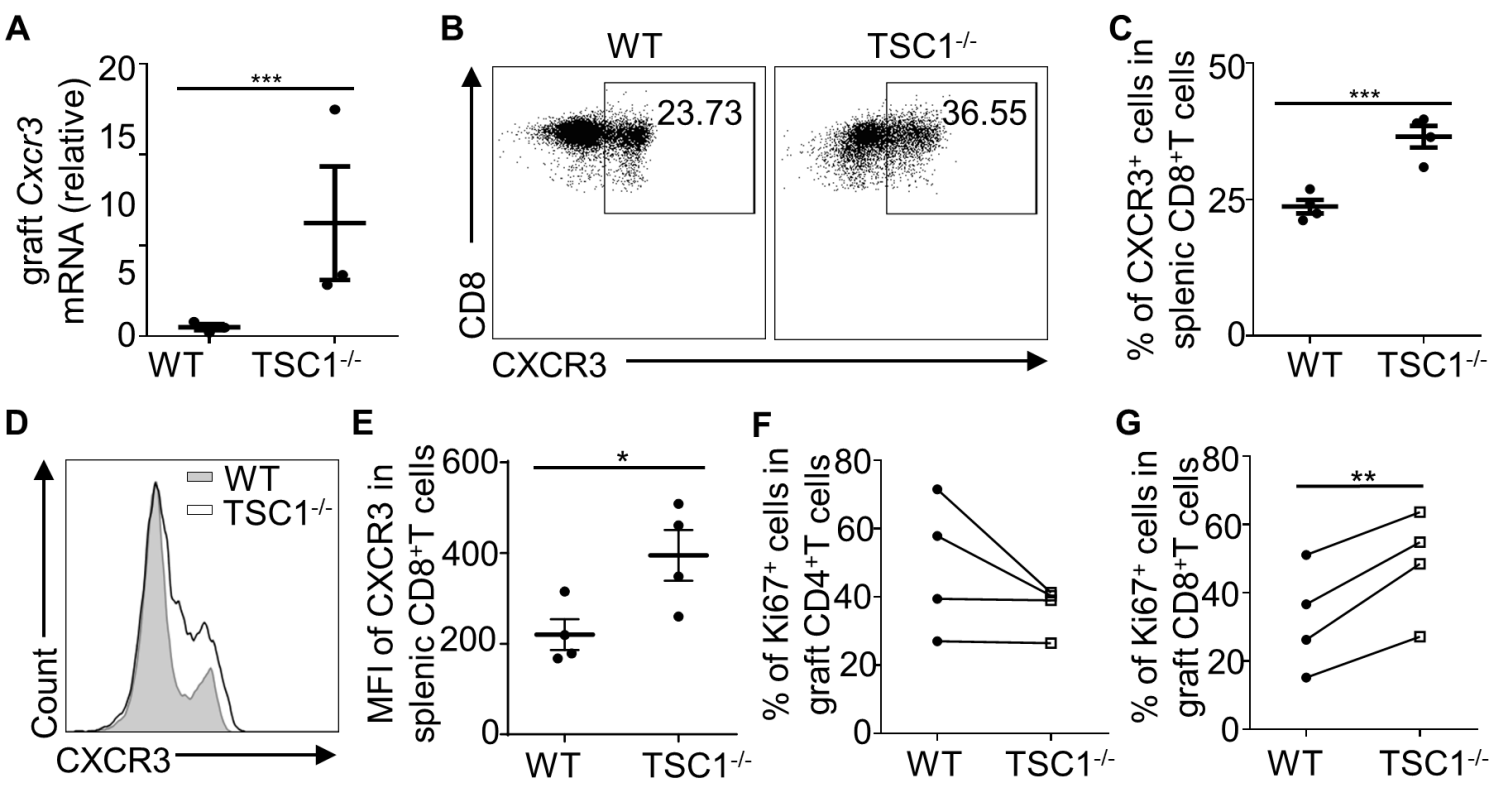
**TSC1-deficiency increases CXCR3 expression and inner-graft proliferation of CD8^+^ T cells**. MHC full-mismatched cardiac allografts from BALB/c mice were transplanted into WT or TSC1^-/-^ mice, and these recipient mice were sacrificed on day 4 post-transplantation. The mRNA levels of *Cxcr3* in the allografts were measured using real-time PCR (A). Mann-Whitney U test; ***P < 0.005, n =3 per group. CXCR3 expression levels in splenic CD8^+^ T cells were determined (B, C, D, and E). Student’s *t*-test; *P < 0.05, ***P < 0.005, n = 4 per group. We also tested t Ki67 expression levels in CD4^+^ (F) and CD8^+^ (G) T cells in allografts. Student’s *t*-test; **P < 0.01, n = 4 per group.

Chemokines are important for T cell allograft infiltration, and CXCR3 has been found to mediate T cell infiltration in allografts[31-33]. As CXCR3 expression levels are upregulated in TSC1-deficient T cells[17], we aimed to determine the expression levels of CXCR3 in alloreactive TSC1^-/-^ CD8^+^ T cells. To this end, we measured the mRNA levels of *Cxcr3* in the allografts on day 4 post-transplantation. *Cxcr3* expression levels were approximately 12.59 times higher in TSC1^-/-^ mice than in WT mice (Fig. 4A). In addition, compared to WT CD8^+^ T cells, TSC1^-/-^ CD8^+^ T cells had a higher percentage of CXCR3^+^ cells and a higher mean fluorescence intensity (MFI) of CXCR3 expression (Fig. 4B-4E). Therefore, TSC1 deficiency increases CXCR3 expression level in alloreactive CD8^+^ T cells, which may promote CD8^+^ T cell allograft migration.

To assess in situ proliferation of TSC1^-/-^ CD8^+^ T cells, allografts in WT or TSC1^-/-^ mice were obtained on day 4 post-transplantation. The percentage of CD4^+^ Ki67^+^ cells in TSC1^-/-^ mice was the same as that in WT mice (Fig. 4F). However, the percentage of CD8^+^ Ki67^+^ cells in TSC1^-/-^ mice was significantly higher than that in WT mice (Fig. 4G). Taken together, these results indicate that TSC1 deficiency enhances CD8^+^ T cell proliferation in allografts. TSC1 deficiency promotes allograft-accumulation of CD8^+^ T cells by enhancing allograft-infiltration and in situ proliferation.

### TSC1 deficiency promotes alloantigen-stimulated proliferation of CD8^+^T cells

**Figure 5.**
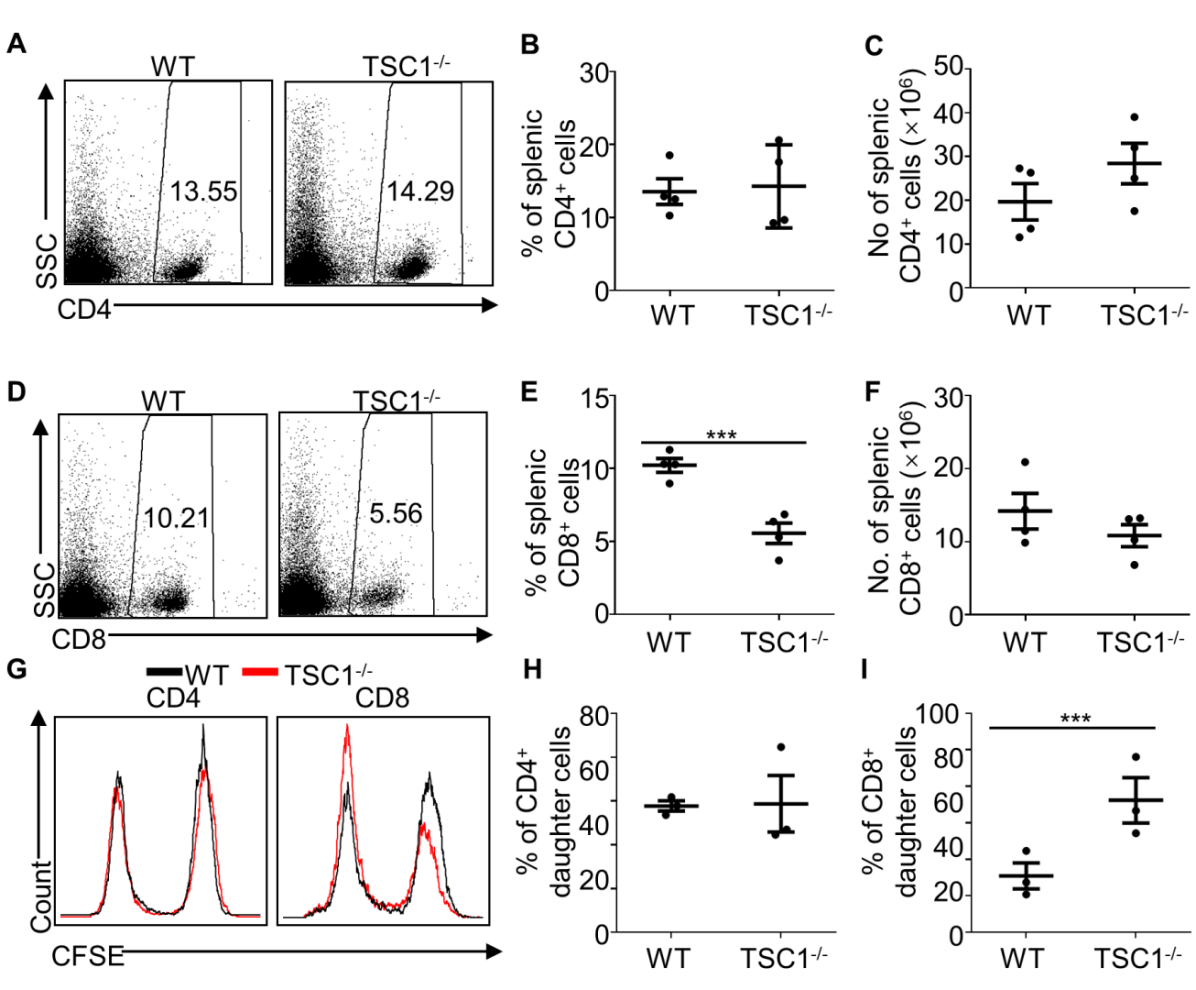
**TSC1 deficiency enhances the proliferation of CD8^+^T cells in vivo after transplantation**. We transplanted the MHC full-mismatched cardiac allografts from BALB/c mice into WT or TSC1^-/-^ mice and determined the percentage and number of CD4^+^ (A, B, and C) and CD8^+^ (D, E and F) T cells on day 4 post-transplantation. Student’s *t*-test; ***P < 0.005, n = 4 per group. In mixed lymphocyte reaction (MLR), we labeled the splenocytes from primary WT or TSC1^-/-^ mice with CFSE and stimulated them with mitomycin C-treated splenocytes from primary BALB/c mice for 72 h in vitro. We then analyzed the proliferation of CD4^+^ and CD8^+^ T cells (G, H, and I). Student’s *t*-test; * *P* < 0.05, n = 3 per group.

We showed that TSC1 deficiency in T cells significantly decreased the number of splenic CD8^+^ T cells but not CD4^+^ T cells in primary mice[13] (Supplementary Fig. 1). On day 4 post-transplantation, the percentage and number of splenic CD4^+^T cells were comparable between WT and TSC1^-/-^ mice (Fig. 5A-5C). Although the percentage of splenic CD8^+^ T cells in WT mice was lower than that in TSC1^-/-^ mice (*P* < 0.05, Fig. 5D, 5E), the number of splenic CD8^+^ T cells was comparable between WT and TSC1^-/-^ mice (Fig. 5F). These results suggest that TSC1 deficiency enhances the proliferation of splenic CD8^+^ T cells after alloantigen stimulation in vivo. Mixed lymphocyte reaction (MLR) was performed to further determine whether TSC1 deficiency enhanced CD8^+^ T cell proliferation after alloantigen stimulation. We found that TSC1 deficiency did not change the proliferation of CD4^+^ T cells but enhanced the CD8^+^ T cell proliferation after alloantigen-stimulation in vitro (Fig. 5G-5I). Hence, TSC1 deficiency enhances the proliferation of CD8^+^ T cells after alloantigen stimulation.

### TSC1 deficiency enhances the functional molecule production by CD8^+^T cells in acute allograft rejection

**Figure 6.**
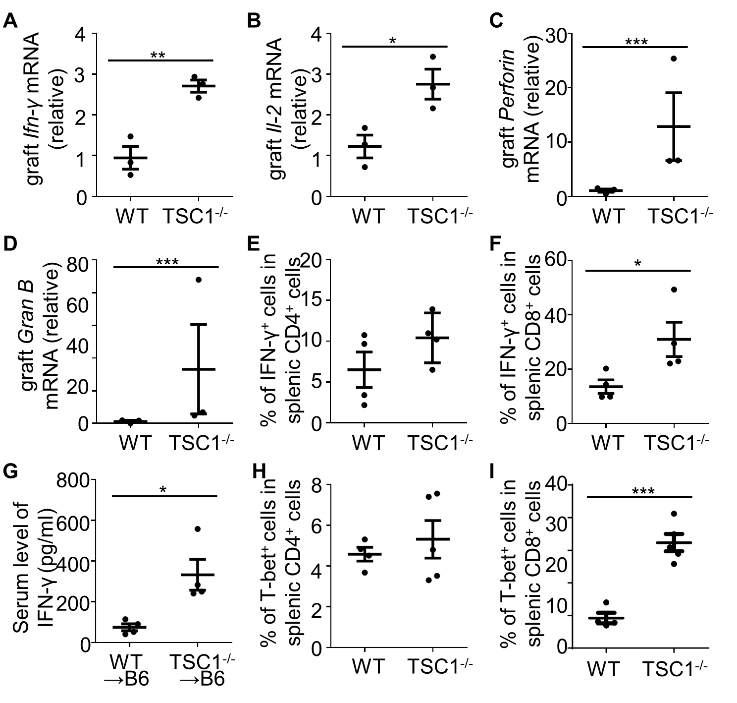
**TSC1 deficiency upregulates the functional molecule production by CD8^+^ T cells**. We implanted allografts into WT or TSC1^-/-^ mice, sacrificed them on day 4 post-transplantation, and determined the mRNA levels of *Ifn-γ* (A), *Il-2* (B), *perforin* (C), and *granzyme B* (D) in allografts via real-time PCR. (**A**) and (B) were determined using the Student’s *t*-test; (**C**) and (D) were determined using the Mann-Whitney U test; *P < 0.05, **P < 0.01, ***P < 0.005, n = 3 per group. On day 4 post-transplantation, IFN-γ production by splenic CD4^+^ and CD8^+^ T cells was tested after PMA, ionomycin, and Golgistop treatment for 4-5 h ex vivo (E and F). Student’s *t*-test; * *P* < 0.05, n = 4 per group. Serum levels of IFN-γ in recipient B6 mice that were implanted with TSC^-/-^ or WT CD8^+^ T cells before cardiac allograft transplantation were tested on day 3 post-transplantation (G). Student’s *t*-test; * *P* < 0.05, n = 4 per group. We also determined T-bet expression levels of splenic CD4^+^ (H) and CD8^+^ (I) T cells in the allografts from WT or TSC1^-/-^ mice on day 4 post-transplantation. (**G**) and (I) were analyzed using the Student’s *t*-test. (**H**) was analyzed using the Mann-Whitney U test; *P < 0.05, ***P < 0.005, n = 4 per group.

**Figure 7.**
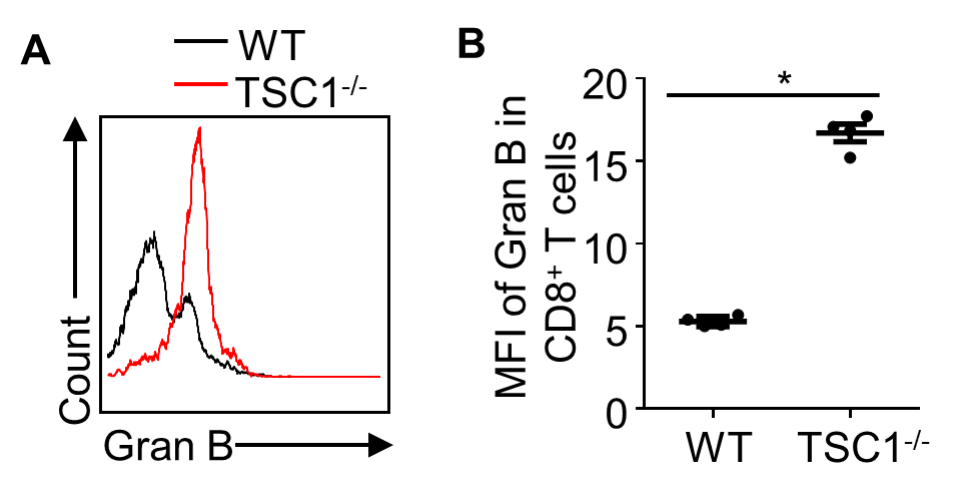
**TSC1 deficiency enhances granzyme B production by CD8^+^ T cells**. In MLR, we tested the granzyme B (Gran B) production by CD8^+^ T cells (A and B). Mann-Whitney U test; *** *P* < 0.005, n = 4 per group.

To determine the effect of TSC1 on effector molecule production by alloreactive T cells, mRNA levels of key effector molecules in allografts were determined on day 4 post-transplantation. Compared to the allografts from WT mice, the mRNA expression levels of proinflammatory cytokines, *Ifng* and interleukin 2 (*Il-2*), were approximately 3- and 2-fold higher respectively, in the allografts from TSC1^-/-^ mice (Fig. 6A, B). The cytotoxic factors, perforin (12.85 to 1.08) and granzyme B (33.19 to 1.24), in the allografts of TSC1^-/-^ mice showed approximately 12- and 26-fold higher expression levels, respectively, compared to the allografts of WT mice (Fig. 6C, D). Although splenic TSC1^-/-^CD4^+^ T cells had a similar percentage of IFN-γ^+^ cells to WT CD4^+^ T cells, splenic TSC1^-/-^CD8^+^ T cells had significantly more IFN-γ^+^ cells than WT CD8^+^ T cells (Fig. 6E, F). To further assess the effect of TSC1^-/-^CD8^+^ T cells on IFN-γ production, serum was obtained from recipient mice with pre-transplant WT and TSC1^-/-^CD8^+^ T cell adoptive transfer. The serum level of IFN-γ in heart-grafted mice with TSC1^-/-^CD8^+^ T cell adoptive transfer (349.6 ± 104.2 pg/mL) were about 4.7-fold higher than those in heart-grafted mice with WT CD8^+^ T cell adoptive transfer (74.9 ± 16.85 pg/mL) on day 3 post-transplantation (Fig. 6G). T-bet regulates IFN-γ production in T cells [34, 35]. We analyzed T-bet expression levels in splenic T cells of heart-transplanted mice on day 4 post-transplantation. WT and TSC1^-/-^CD4^+^ T cells had identical T-bet^+^ phenotype, but TSC1^-/-^CD8^+^ T cells had a higher percentage of T-bet^+^ cells than WT CD8^+^ T cells (Fig. 6H, I). In MLR, TSC1^-/-^CD8^+^ T cells produced more granzyme B than WT CD8^+^ T cells after alloantigen stimulation (Fig. 7A, B). Therefore, TSC1 deficiency promotes IFN-γ and granzyme B expression in alloreactive CD8^+^T cells, which may contribute to accelerated cardiac allograft rejection in mice.

### TSC1 deficiency in T cells impairs the effect of rapamycin on allograft survival

**Figure 8.**
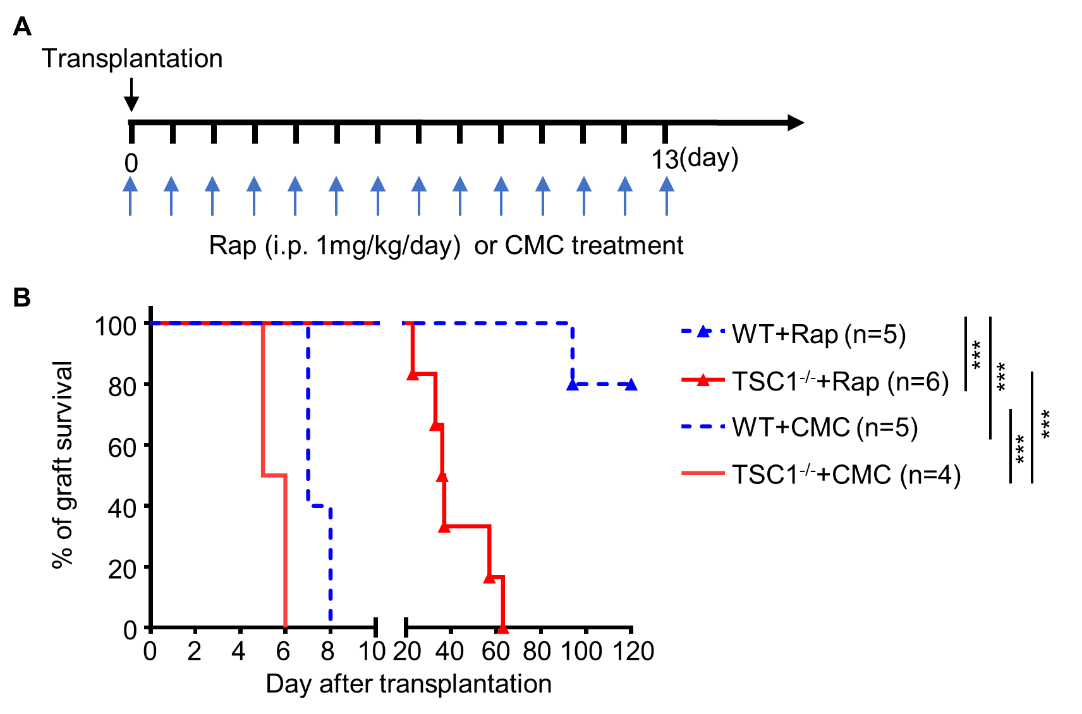
**TSC1 deficiency in T cells impairs the effect of rapamycin on allograft survival**. We transplanted MHC full-mismatched cardiac allografts from BALB/c mice into WT or TSC1^-/-^ mice. Rapamycin or CMC was administered from day 0 to 13 post-transplantation (A), and the survival of allografts was analyzed (B). Log-rank test; ***P < 0.005, n = 4-6 per group. CMC, carboxymethyl cellulose sodium; Rap, rapamycin; i.p., intraperitoneal.

TSC1 deficiency causes increased activation of mTORC1 in T cells[13, 17, 18], and mTOR inhibitors, which have been extensively used to prevent allograft rejection, are used to treat patients with TSC[5, 11, 36]. To illustrate the effects of mTOR inhibitors on allograft survival in mice with TSC1-deficient T cells, WT and TSC1^-/-^ mice were treated with rapamycin or CMC from day 0 to 13 after transplantation (Fig. 8A). Rapamycin treatment induced 80% cardiac allograft survival over long-term (> 120 d; Fig. 8B). Although rapamycin significantly prolonged the allograft survival in TSC1^-/-^ mice (41.50 ± 6.24 d), all cardiac allografts were rejected (Fig. 8B). These results suggest that TSC1^-/-^ recipient mice are resistant to rapamycin-induced long-term survival of cardiac allografts.

## DISCUSSION

A longstanding question in organ transplantation is how to inhibit the alloimmune response and achieve allograft tolerance [37-39]. As the cellular components in the alloimmune response, alloreactive CD8^+^ T cells are the major effector cell population responsible for impairing allografts after the initiation of acute allograft rejection [40, 41]. However, the immunological mechanisms of CD8^+^ T cells in transplant response are still limited. In this study, we determined that TSC1 regulates the proliferation, graft accumulation, and response capacities of alloreactive CD8^+^ T cells during acute allograft rejection.

TSC1 deficiency impairs CD8^+^ T cell responses to bacterial infections [20, 21]. We [13] and others [18, 19] have previously found that TSC1 depletion in T cells decreases the number of peripheral CD8^+^T cells in mice. Thus, we assumed that the impaired function of CD8^+^T cells and the lymphopenic environment may be a beneficial for transplant outcomes. Unexpectedly, allografts in TSC1^-/-^ mice exhibited accelerated acute rejection. Notably, more CD8^+^ T cell graft accumulation was found in the cardiac allografts of TSC1^-/-^ mice. CXCR3 is suggested to mediate the graft infiltration of T cells during the transplant response and negatively affect allograft survival [31-33]. In this study, TSC1 deficiency resulted in enhanced expression levels of CXCR3 in CD8^+^ T cells during the transplant response. This result is in line with a previous report showing that CXCR3 is highly expressed in TSC1-deficient T cells [17]. In addition to infiltration, in situ proliferation is another method of T-cell accumulation [29, 42]. Compared with allograft-infiltrated WT CD8^+^ T cells, we found that TSC1 deficiency enhanced Ki67 expression levels in allograft-infiltrated CD8^+^ T cells. Thus, both enhanced infiltration and proliferation of alloreactive CD8^+^ T cells in TSC1-deficient recipient mice may contribute to accelerated cardiac allograft rejection.

TSC1 deficiency significantly decreases the number of splenic CD8^+^T cells in primary mice [13, 17, 19]. However, the number of CD8^+^ T cells in the spleens of TSC1^-/-^ mice is comparable to WT mice on day 4 post-transplantation. The regulatory effect of TSC1 on CD8^+^ T cell proliferation was further demonstrated in MLR, which is consistent with previous results that TSC1-deficiency increased the proliferation of CD8^+^ T cells [17, 18]. These data suggest that TSC1 deficiency can enhance the proliferation of CD8^+^ T cells in allograft transplant recipients, and enhanced T cells proliferation is detrimental to allograft survival [43]. However, some colleagues demonstrated that TSC1 deficiency did not change the proliferation of CD8^+^ T cells *in vitro*, but inhibited their proliferation in vivo in a bacterial infection model [21]. TSC1 deficiency had no effect on proliferation of CD8^+^ T cells after anti-CD3 and anti-CD28 stimulation but enhances the proliferation of CD8^+^ T cells by anti-CD3 stimulation *in vitro* [22]. This inconsistency may be due to the use of different models. In addition, our results showed that the deficiency of TSC1 did not affect the proliferation of CD4^+^ T cells after alloantigen stimulation and that TSC1^-/-^ mice and WT mice also had comparable splenic CD4^+^ T cells before and after transplantation. In line with this study, our previous study demonstrated that TSC1 deficiency did not affect the homeostasis of CD4^+^ T cells [13] and the proliferation of CD4^+^ T cells *in vitro* [22].

IFN-γ and granzyme B are critical factors for transplant rejection in alloreactive CD8^+^ T cells [44, 45]. We demonstrated that TSC1 deficiency significantly enhanced IFN-γ and granzyme B production in alloreactive CD8^+^ T cells and increased the serum levels of IFN-γ in recipient mice. IFN-γ production by splenic CD4^+^ T cells was not affected by TSC1 deficiency in this study, which is consistent with the reported results that TSC1 deficiency does not change IFN-γ production by CD4^+^ T cells with anti-CD3 and anti-CD28 stimulation [22]. However, TSC1 deficiency inhibits IFN-γ expression in CD8^+^ T cells after bacterial antigen-stimulation [20, 21] and enhances IFN-γ production by CD4^+^ T cells and Th1 differentiation in mice with induced colitis [46]. T-bet is essential for alloreactive CD8^+^T cells to express IFN-γ, CXCR3, granzyme B, and Ki67 [47]. Researchers found that TSC1 deficiency inhibits T-bet expression in naïve CD8^+^ T cells but not in CD8^+^ T cells after bacterial antigen stimulation *in vivo* [21]. In fact, the expression levels of T-bet were consistent with the changes in IFN-γ expression levels observed in this study. T-bet expression levels were upregulated in alloreactive CD8^+^ T cells, but not in CD4^+^ T cells in recipient mice. In line with our results, other researchers have demonstrated that TSC1 deficiency enhances the granzyme B expression levels in antigen-specific CD8^+^ T cells [20]. Interestingly, T-bet is also required for CD8^+^ T-cell survival [47]. Taken together, TSC1 deficiency enhances T-bet, IFN-γ, and granzyme B expression levels of CD8^+^ T cells, and the role of T-bet in the regulation of TSC1 in alloreactive CD8^+^ T cells should be further studied.

Considering the effects of mTORC1 inhibitors on TSCs and organ transplantation [5, 11], mTORC1 inhibitors may be the best choice for immunosuppressive therapy in patients with TSC after organ transplantation [36]. In fact, growing evidence has suggests that TSC1 can regulate some of the biological actions of immune cells by mTORC1-independent pathways. TSC1 regulated the apoptosis of T cells [13, 17] and dendritic cells [48], partly via Bim-dependent pathways, and M1 polarization of macrophages via Ras/GTPase-/Raf/MEK/ERK pathway [15]. In this study, rapamycin treatment significantly prolonged allograft survival in both TSC1^-/-^ mice and WT mice, but rapamycin was less effective in TSC1^-/-^ mice, and TSC1-deficient T cells may be detrimental to the long-term survival of cardiac allografts in mice. Therefore, the effect of rapamycin following the conventional usage may be limited, and a better single or combination therapy with an mTOR inhibitor may be essential for the long-term improved outcome of patients with TSC after organ transplantation.

In conclusion, TSC1 deficiency accelerates acute rejection by enhancing the alloreactivity of CD8^+^ T cells. During alloimmune response, TSC1-deficient CD8^+^ T cells enhance graft-accumulation, proliferation, and effector molecule production. Furthermore, TSC1-deficient T cells show a high activation of mTORC1, however, TSC1 deficiency impairs the effect of mTOR inhibitor rapamycin on allograft survival.

## Supplementary Materials

The Supplementary data can be found online at: www.aginganddisease.org/EN/10.14336/AD.2022.0224.


