# Supplementary material for: Absence of TSC1 Accelerates CD8+ T cell-mediated Acute Cardiac Allograft Rejection
Source: Aging Dis. 2022 Oct 1;13(5):1562–75. doi: 10.14336/AD.2022.0224 (PMC9466980; doi:10.14336/AD.2022.0224)
Supplement: Supplementary file 1 [file AD-13-5-1562-s.pdf]

SUPPLEMENTARY DATA

Absence of TSC1 Accelerates $CD8^+$ T cell-mediated Acute Cardiac Allograft Rejection

Liang Tan[1,2,#], Yanan Xu[3,#], Gongbin Lan[1,2,#], Hongxia Wang[4,#], Zhanfeng Liang[3], Zhaoqi Zhang[3], Qianchuan Tian[3], Yangxiao Hou[3], Yong Zhao[3,5], Xubiao Xie[1,2,*]

© 2022. Tan L. et al. Published online at http://www.aginganddisease.org/EN/10.14336/AD.2022.0224

SUPPLEMENTARY DATA

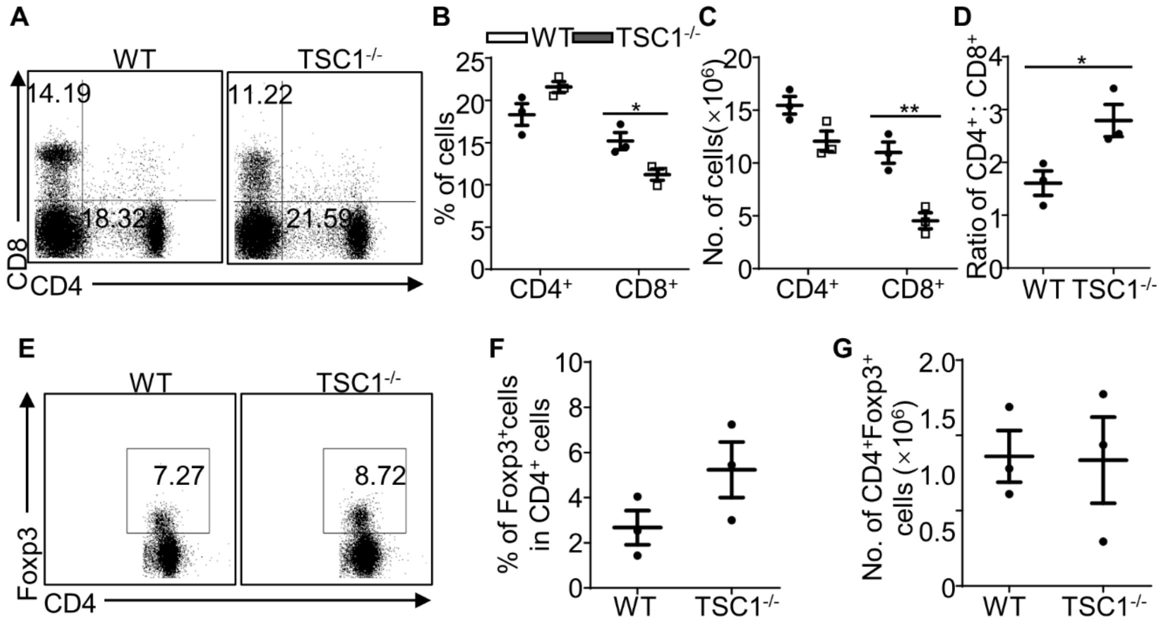

Supplementary Figure 1. TSC1 deficiency in T cells decreased peripheral CD8$^+$T cells in primary mice. We sacrificed the primary WT or TSC1$^{-/-}$ mice, and analyzed the splenic T cells (A, B, C and D) and Foxp3$^+$CD4$^+$ T cells (E, F and G). Student's t-test; * $P < 0.05$, ** $P < 0.05$, n = 3 per group.

© 2022. Tan L. et al. Published online at http://www.aginganddisease.org/EN/10.14336/AD.2022.0224
